# Association of Variants at *UMOD* with Chronic Kidney Disease and Kidney Stones—Role of Age and Comorbid Diseases

**DOI:** 10.1371/journal.pgen.1001039

**Published:** 2010-07-29

**Authors:** Daniel F. Gudbjartsson, Hilma Holm, Olafur S. Indridason, Gudmar Thorleifsson, Vidar Edvardsson, Patrick Sulem, Femmie de Vegt, Frank C. H. d'Ancona, Martin den Heijer, Leifur Franzson, Thorunn Rafnar, Kristleifur Kristjansson, Unnur S. Bjornsdottir, Gudmundur I. Eyjolfsson, Lambertus A. Kiemeney, Augustine Kong, Runolfur Palsson, Unnur Thorsteinsdottir, Kari Stefansson

**Affiliations:** 1 deCODE genetics, Reykjavik, Iceland; 2 Division of Nephrology, Department of Medicine, Landspitali University Hospital, Reykjavik, Iceland; 3 Children's Medical Center, Landspitali University Hospital, Reykjavik, Iceland; 4 Faculty of Medicine, School of Health Sciences, University of Iceland, Reykjavik, Iceland; 5 Department of Epidemiology, Biostatistics, and Health Technology Assessment, Radboud University Nijmegen Medical Centre, Nijmegen, The Netherlands; 6 Department of Urology, Radboud University Nijmegen Medical Centre, Nijmegen, The Netherlands; 7 Department of Endocrinology, Radboud University Nijmegen Medical Centre, Nijmegen, The Netherlands; 8 Department of Genetics and Molecular Medicine, Landspitali University Hospital, Reykjavik, Iceland; 9 The Laboratory in Mjodd, Reykjavik, Iceland; University of Oxford, United Kingdom

## Abstract

Chronic kidney disease (CKD) is a worldwide public health problem that is associated with substantial morbidity and mortality. To search for sequence variants that associate with CKD, we conducted a genome-wide association study (GWAS) that included a total of 3,203 Icelandic cases and 38,782 controls. We observed an association between CKD and a variant with 80% population frequency, rs4293393-T, positioned next to the *UMOD* gene (GeneID: 7369) on chromosome 16p12 (OR = 1.25, *P* = 4.1×10^−10^). This gene encodes uromodulin (Tamm-Horsfall protein), the most abundant protein in mammalian urine. The variant also associates significantly with serum creatinine concentration (SCr) in Icelandic subjects (N = 24,635, *P* = 1.3×10^−23^) but not in a smaller set of healthy Dutch controls (N = 1,819, *P* = 0.39). Our findings validate the association between the *UMOD* variant and both CKD and SCr recently discovered in a large GWAS. In the Icelandic dataset, we demonstrate that the effect on SCr increases substantially with both age (*P* = 3.0×10^−17^) and number of comorbid diseases (*P* = 0.008). The association with CKD is also stronger in the older age groups. These results suggest that the *UMOD* variant may influence the adaptation of the kidney to age-related risk factors of kidney disease such as hypertension and diabetes. The variant also associates with serum urea (*P* = 1.0×10^−6^), uric acid (*P* = 0.0064), and suggestively with gout. In contrast to CKD, the *UMOD* variant confers protection against kidney stones when studied in 3,617 Icelandic and Dutch kidney stone cases and 43,201 controls (OR = 0.88, *P* = 5.7×10^−5^).

## Author Summary

Chronic kidney disease (CKD) is a common condition that is associated with substantial morbidity and mortality and has been recognized as a major public health problem worldwide. Common causes of CKD include hypertension, diabetes, and inflammatory disorders. Previous studies have shown a significant genetic contribution to kidney disease and a recent genome-wide association study yielded a variant in the *UMOD* gene that affects the risk of CKD. Here, we replicate the association between *UMOD* and CKD in an independent analysis. We also demonstrate for the first time an interaction between the *UMOD* variant and age that suggests that this variant may adversely affect the aging kidney and its adaptation to age-related risk factors of kidney disease, such as hypertension and diabetes. Furthermore, we show that the *UMOD* variant that affects risk of CKD also provides protection against kidney stone disease.

## Introduction

Chronic kidney disease (CKD) is a common disorder that can progress to kidney failure and is associated with an increased risk of cardiovascular disease and mortality [1], [2]. The cause of CKD is not always known and frequently appears multifactorial with hypertension (HTN) and diabetes mellitus (DM) being the most important causes [3]–[6]. Other causes include intrinsic kidney disorders, atherosclerosis and nephrotoxic drugs [7], [8]. Studies also indicate a dramatic increase in the prevalence of CKD with advancing age [9], [10]. With greater lifespan, the burden of CKD is thus steadily rising in the Western world [11], resulting in a substantial impact on the health care system [12].

Previous studies have suggested a genetic contribution to the risk of kidney disease. Heritability estimates of serum creatinine (SCr) and estimated glomerular filtration rate based on SCr (eGFRcrea), both common measures of kidney function, have been reported as 29% and 33%, respectively [13]. Recently, Köttgen et al. [14] published the first genome-wide association study (GWAS) on eGFRcrea, eGFR based on cystatin C (eGFRcys), another measure of kidney function, and CKD, reporting significant association with eGFRcrea at three loci (*UMOD*, *SHROOM3* (GeneID: 57619) and *GATM-SPATA5L1* (GeneIDs: 2628 and 79029)), with eGFRcys at two loci (*CST3-CST9* (GeneIDs: 1471 and 128822) and *STC1* (GeneID: 6781)) and with CKD at one locus (*UMOD*) [14].

With the aim of discovering sequence variants that associate with kidney function, we conducted a GWAS in a total of 3,203 Icelanders with CKD and 38,782 controls and in 24,635 Icelandic and 1,819 Dutch subjects with SCr information. We found a sequence variant at the *UMOD* locus that associates with both CKD and SCr at a genome wide-significant (GWS) level, providing an independent replication of the result by Köttgen et al [14]. We also show that this variant interacts with age-related increase in SCr levels with little or no effect on SCr before the age of 50 years, followed by a rapidly growing effect with increasing age. We demonstrate that this variant associates significantly with serum urea, uric acid and suggestively with gout. In contrast to the deleterious effect on kidney function, the variant confers protection against kidney stone disease.

## Results/Discussion

### Genome-wide association of variants at the *UMOD* locus with CKD and SCr

A GWAS of 2.5 million SNPs, either directly genotyped (Illumina HumanHap300 or HumanHapCNV370 bead chips) or imputed based on the HapMap CEU samples [15], was performed on a sample set of 2,903 Icelanders with CKD (see Materials and Methods for sample set description) and 35,818 controls and also on 22,256 Icelandic subjects with SCr information (See QQ-plots in Figure S1 and Figure S2). The Icelandic SCr measurements came from two laboratories; the Laboratory in Mjodd, a private outpatient laboratory, and the Clinical Biochemistry Laboratory of Landspitali University Hospital (LUH), serving both inpatients and outpatients. These subjects had 5.2 SCr measurements on average (geometric mean) and we used the median SCr value for each individual in the subsequent analysis. The SCr values from the two Icelandic laboratories showed similar dependence on age and sex but there was clearly a trend towards higher SCr in the hospital laboratory compared with the outpatient laboratory (Figure S3).

The GWAS on CKD and SCr both yielded several SNPs in high linkage disequilbrium (LD) on chromosome 16p12 with GWS (*P*<5×10^−8^) association to increased risk of CKD and elevated SCr. For both phenotypes, this signal is tagged by rs4293393-T (Table 1 and Table 2). For CKD, the odds ratio (OR) conferred by rs4293393-T was 1.25 (95% CI = 1.16–1.34) with a corresponding *P* value of 6.2×10^−9^. In an attempt to replicate this finding, rs4293393 was typed in additional 300 Icelandic subjects with CKD and 2,964 controls. The association was nominally significant in the replication sample set and the effect size consistent with the initial observation (Table 1). The combined OR for rs4293393-T in the two Icelandic CKD sample sets was 1.25 (95% CI = 1.17–1.35) and *P* = 4.1×10^−10^. The association between SCr and rs4293393-T on 16p12 was very strong with an effect of 1.86 µmol/L per allele carried and *P* = 6.7×10^−20^ (Table 2). To follow up on these results, we genotyped rs4293393 in 2,379 additional Icelanders with SCr information, significantly replicating the initially observed effect (*P* = 1.4×10^−5^, Table 2). Analysis of the combined Icelandic datasets showed a strong GWS association between rs4293393-T and elevated SCr (effect = 1.93 µmol/L per allele, 95% CI = 1.55–2.31 µmol/L; *P* = 1.3×10^−23^). Our findings provide an independent replication of the recently reported results by Köttgen et al [14] of an association of this 16p12 locus with CKD and eGFRcrea. The strongest SNP associations outside the *UMOD* region on chromosome 16p12 are shown in Table S1 (for CKD) and Table S2 (for SCr), respectively.

**Table 1:** Association of rs4293393-T with chronic kidney disease (CKD).

CKD	N		Frequency			
population	case	control	case	control	OR (95% CI)	*P*
Icelandic discovery	2,903	35,818	0.831	0.798	1.25 (1.16, 1.34)	6.2×10^−9^
Icelandic replication	300	2,964	0.840	0.798	1.33 (1.05, 1.68)	0.019
Icelandic combined	3,203	38,782	0.832	0.798	1.25 (1.17, 1.35)	4.1×10^−10^
YOB≥1950	143	38,782	0.811	0.798	1.09 (0.81, 1.46)	0.57
1950>YOB≥1940	355	38,782	0.821	0.798	1.16 (0.96, 1.41)	0.12
1940>YOB≥1930	1,029	38,782	0.834	0.798	1.28 (1.14, 1.43)	3.1•10^−5^
1930>YOB≥1920	1,242	38,782	0.838	0.798	1.31 (1.18, 1.46)	4.1•10^−7^
1920>YOB	434	38,782	0.825	0.798	1.19 (1.00, 1.42)	0.045

Association of rs4293393-T with CKD in Icelandic subjects. Association is shown for the discovery sample set, the replication sample set, the combined Icelandic sample, and finally, for the combined sample stratified by year of birth (YOB) as a proxy for age at onset.

**Table 2:** Association of rs4293393-T with serum creatinine concentration (SCr).

SCr sample	N	Effect (95% CI)	P
Icelandic discovery	22,256	1.86 (1.46, 2.25)	6.7×10^−20^
Icelandic replication	2,379	2.68 (1.47, 3.89)	1.4×10^−5^
Icelandic combined	24,635	1.93 (1.55, 2.31)	1.3×10^−23^
Dutch replication	1,819	0.38 (−0.48, 1.25)	0.39

Association of rs4293393-T with SCr in Icelandic and Dutch subjects. Association is shown for the discovery sample set, the Icelandic and Dutch replication sample sets and the combined Icelandic sample. The population frequency of rs4293393-T is 0.80 in Iceland and 0.81 in the Netherlands. Effects are given in µmol/L.

For further assessment, we tested rs4293393 in 1,819 Dutch subjects with SCr information. These were healthy population-based controls (see Materials and Methods for sample set description) with SCr values substantially different from the Icelandic measurements, generally showing lower values and much less variability (Figure S3). Interestingly, no association was observed in the 1,819 healthy Dutch subjects (effect = 0.38 µmol/L, 95%CI = −0.48–1.25 µmol/L; *P* = 0.39) (Table 2). Significant heterogeneity was observed between the SCr association results for the Icelandic and Dutch populations (I^2^ = 90.4%, *P* = 0.0013).

**Figure 1:**
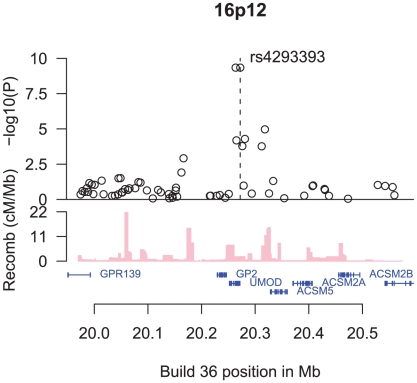
An overview of the region around rs4293393. Shown are the −log_10_ association *P* values of SNPs in the region with CKD (black circles), the SNPs' build 36 coordinates, the genes in the region and their exons (in blue), and recombination rates in centimorgans (cM) per megabase (Mb) (pink histogram).

The SNP rs4293393 is located 550 basepairs upstream of *UMOD*, encoding uromodulin, also known as the Tamm-Horsfall protein (Figure 1). The protein is a glycosylphosphatidylinositol (GPI)-anchored glycoprotein, exclusively expressed in the thick ascending loop of Henle [16] and distal convoluted tubule [17] of the mammalian kidney. It is the most abundant protein in urine of healthy individuals, where it is present in a highly aggregated state [18], [19]. While the exact physiological function of uromodulin remains to be elucidated, it has the capacity to bind to a variety of ligands. It has been reported to prevent bacteria from adhering to human kidney cells [20] and to inhibit calcium oxalate crystallization [21]. It may also have a role in ion transport and immunological processes [22], [23]. *UMOD* knockout mice have been shown to have decreased creatinine clearance [24] and predilection for both urinary tract infections [25] and calcium oxalate stone formation [26].

The rs4293393 variant is in perfect LD in the HapMap CEU samples [15] with a synonymous SNP in *UMOD*, rs13335818, coding for V264V (D′ = 1.0, *r* ^2^ = 1.0). The same perfect correlation between rs4293393 and rs13335818 was observed in a set of 3,364 Icelanders (D′ = 1.0, *r* ^2^ = 1.0). Both rs4293393 and rs13335818 are also in perfect correlation with rs12917707 (D′ = 1.0, *r* ^2^ = 1.0 for both markers in the HapMap CEU samples [15]) near the *UMOD*, the variant reported by Köttgen et al [14] to associate with both CKD and eGFRcrea with similar effect, indicating that these SNPs are tagging the same signal. As rs4293393 is on the Illumina 300/370K chips we used for direct genotyping, we refer to rs4293393 in the remainder of this article.

### The effect of the *UMOD* variant on SCr is age-dependent

**Figure 2:**
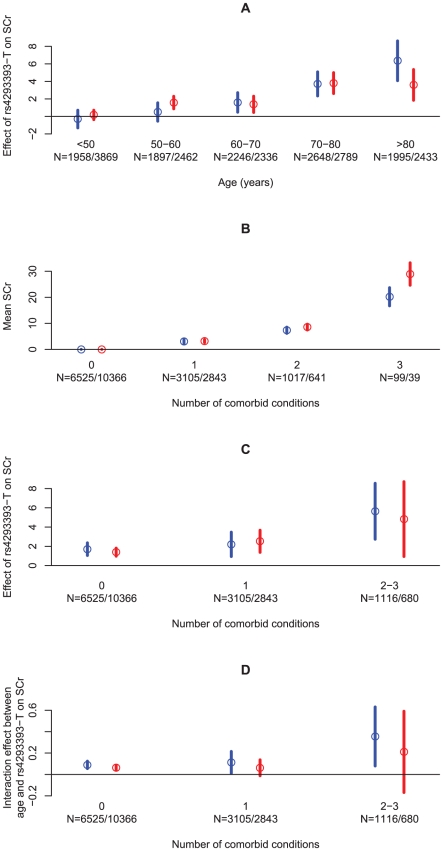
An overview of the effect of age and the number of comorbid conditions on SCr levels, directly and through the rs4293393-T allele count. (A) The effect of rs4293393-T on SCr stratified on age and sex. (B) The mean SCr stratified by the number of comorbid conditions and sex, compared to the mean SCr in those without any comorbid conditions. (C) The effect of rs4293393-T on SCr stratified by the number of comorbid conditions and sex. (D) The interaction effect between age and rs4293393-T allele count on SCr stratified by the number of comorbid conditions and sex. The circles give the point estimates and the vertical lines show their 95% confidence intervals. Estimates and confidence intervals are given in blue for males and red for females. Sample sizes (N) are given for each strata for males and females, respectively. Effects are given in µmol/L in (A–C) and µmol/L/year in (D).

Given that SCr varies substantially with both age and sex, we tested for an interaction between the effect of rs4293393-T and effects of age and sex on SCr. No interaction was found between the *UMOD* variant and sex (*P* = 0.41). In contrast, a strong interaction was observed between the *UMOD* variant and age in the Icelandic sample set (*P* = 3.0×10^17^). On average, SCr increased by an additional 0.09 µmol/L per year per allele of rs4293393-T (95% CI = 0.07–0.11). In order to visualize this interaction, we stratified our Icelandic samples based on age and sex and tested for association within each stratum (Figure 2A). Interestingly, rs4293393-T has little or no effect on SCr before the age of 50 years, but thereafter the effect increases rapidly with advancing age, especially around 70 years. Thus, the variant does not affect SCr in young individuals but rather how SCr increases with age. We note that due to the relatively short time span in which the SCr data were collected there is an inherent confounding between age and generation in our study, which will require further investigation to resolve. Similar interaction between the *UMOD* variant, age and CKD was also observed when the association analysis for CKD was stratified by year of birth used here as a proxy for age of onset (Table 1).

Although it is well known that kidney function declines with age, GFR has been shown to decrease more slowly with senescence in healthy individuals than previously thought [3]–[5], [11]. Comorbid conditions that increase in frequency with aging, including HTN, DM, atherosclerosis and heart failure are, however, increasingly recognized as important contributors of age-related decline in kidney function [3], [4], [6]–[8]. We thus proceeded to investigate whether the age effect observed in carriers of the *UMOD* variant is influenced by interaction with age-related risk factors for decline in kidney function.

### *UMOD*-associated increase in SCr with age is affected by the number of comorbid conditions present

As HTN, type 2 DM and atherosclerosis are all well recognized age-dependent risk factors for CKD [3], [4], [6]–[8], the association analysis was repeated after stratifying the SCr data based on these conditions. Incomplete information regarding history of HTN (5,705 cases), type 2 DM (1,422 cases) and myocardial infarction (MI, 2,551 cases) was available for the Icelandic SCr sample set. In parallel with previous studies, the rate of increase in SCr with age was significantly higher in individuals with HTN than in individuals without this diagnosis (effect = 0.23 µmol/L/year, 95% CI = 0.19–0.26 µmol/L/year; *P* = 2.9×10^−35^). Similar results were obtained for type 2 DM (effect = 0.26 µmol/L/year, 95% CI = 0.19–0.34 µmol/L/year; *P* = 1.1×10^−11^) and MI (effect = 0.36 µmol/L/year, 95% CI = 0.30–0.42 µmol/L/year; *P* = 1.4×10^−32^) as well as the number of comorbid conditions (Figure 2B). We also found that the effect of rs4293393-T on SCr increases with the number of comorbid conditions present (Figure 2C).

To further assess the relationship between genotype, age and risk factors for reduced kidney function, we investigated the effect of the rs4293393-T allele count on the increase in SCr with age stratifying on HTN and type 2 DM. A trend was observed for a higher rate of increase in SCr with age and rs4293393-T allele count in individuals with HTN compared to those without a diagnosis of HTN (*P* = 0.077) as well as in those with type 2 DM compared to those without (*P* = 0.063). In other words, the age-related increase in SCr levels appears to be greater in rs4293393-T carriers that have either HTN or type 2 DM than in carriers who do not have these risk factors. However, an age effect was still observed after accounting for these age-related risk factors. Furthermore, we also observed a significantly higher rate of SCr increase with age and rs4293393-T allele count stratifying on the number of comorbid conditions present (*P* = 0.0080) (Figure 2D).

To determine whether rs4293393-T influenced kidney function by directly affecting known risk factors, we tested the association of rs4293393-T in well powered Icelandic case-control sets of HTN, MI, stroke, and type 2 DM (Table S3). A weak nominally significant association of rs4293393-T with increased risk of HTN was observed (OR = 1.07, 95% CI = 1.01–1.12; *P* = 0.014), but not with the other diseases tested. These data demonstrate that the effect of rs4293393-T on kidney function is not mediated through increased risk of these comorbid conditions, but rather suggest that the variant may affect the vulnerability of the kidney to these risk factors.

These findings, demonstrating not only the effect of age on *UMOD*-associated increase in SCr but also the effect of age-related comorbid conditions, may explain why no association was observed between rs4293393-T and kidney function in the Dutch sample set of healthy population-based subjects with much lower SCr values and of much less variability than observed in the Icelandic samples (Figure S3).

### Association of the *UMOD* variant with serum urea

Urea is another small solute that is commonly used to assess renal function together with SCr. The correlation between SCr and serum urea in our data was 58%. We tested for association between rs4293393-T and serum urea in an Icelandic sample set that had urea measurements performed at the Laboratory in Mjodd (N = 4,084) and found significant association with increased serum urea concentration (effect = 0.36 mg/dL, 95% CI = 0.23–0.50 mg/dL; *P* = 1.0×10^−6^).

### Association of the *UMOD* variant with uric acid and gout

In humans, rare mutations in the *UMOD* gene that cause accumulation of abnormal uromodulin in the nephron and reduced urinary excretion of the normal protein [27] have been associated with two autosomal dominant kidney diseases with overlapping clinical features, medullary cystic kidney disease and familial juvenile hyperuricemic nephropathy [28]. These disorders are characterized by hyperuricemia, gout and progressive renal failure due to tubulointerstitial nephropathy. Given the link between *UMOD* and hyperuricemia, we tested rs4293393-T in Icelandic subjects with serum uric acid values from the Laboratory in Mjodd (N = 6,583) and observed significant association (effect = 6.1, 95% CI = 1.7–10.4; *P* = 0.0064). We then tested for association with gout in a set of 377 Icelandic cases and 39,916 controls (see Materials and Methods for sample set description) and found a suggestive association (OR = 1.17, 95% CI = 0.97–1.41; *P* = 0.097). These data contrast the work of Köttgen et al [14] that neither detected association with serum uric acid nor gout.

### Reduced risk of kidney stone formation in carriers of the *UMOD* CKD risk variant

As uromodulin is thought to prevent the formation of calcium-containing kidney stones [21], we tested rs4293393 in a sample set of 1,689 Icelandic patients with radiopaque kidney stones and 37,076 Icelandic population controls. We observed a significant association between rs4293393-T and reduced risk of kidney stones (OR = 0.88, 95% CI = 0.81–0.96; *P* = 0.0053). In an attempt to replicate this observation, we genotyped rs4293393 in two additional sample sets of European ancestry, one from Iceland (1,271 cases and 3,177 controls) and the other from the Netherlands (701 cases and 2,948 controls) (Table 3). The effect size in both replication samples is consistent with the initial observation and the association is significant in the combined replication samples (OR = 0.89, 95% CI = 0.81–0.97; *P* = 0.0089). There was no correlation between the effect size and year of birth of the kidney stone patients (Table S4).

**Table 3:** Association of rs4293393-T with kidney stones.

Kidney stone	N		Frequency			
population	case	control	case	control	OR (95% CI)	*P*
Icelandic discovery	1,689	37,076	0.781	0.801	0.88 (0.81, 0.96)	0.0053
Icelandic replication	1,271	3,177	0.786	0.801	0.91 (0.81, 1.03)	0.13
Icelandic combined	2,916	40,253	0.782	0.801	0.89 (0.83, 0.95)	0.00075
Dutch replication	701	2,948	0.782	0.810	0.85 (0.73, 0.98)	0.022
Combined	3,617	43,201	-	-	0.88 (0.83, 0.94)	5.7×10^−5^

Association of rs4293393-T with kidney stone disease in Icelandic and Dutch subjects. Association is shown for the discovery sample set, the Icelandic and Dutch replication sample sets, the combined Icelandic sample, and all the sample sets combined.

### Replication of the *SHROOM3* and *GATM-SPATA5L1* eGFRcrea loci and the *STC1* and *CST3-CST9* eGFRcys loci

Köttgen et al [14] reported on variants at additional loci with GWS association to eGFRcrea (*SHROOM3* and *GATM-SPATA5L1*) or eGFRcys (*STC1* and *CST3-CST9*). We tested these variants in our Icelandic datasets, including a small sample set with cystatin C measurements (Table 4). The association with SCr replicated for both the *SHROOM3* and *GATM-SPATA5L1* SNPs (*P* = 0.00057 and *P* = 0.0067, respectively) and the association with cystatin C replicated for the *CST3-CST9* SNP but not the *STC1* SNP (*P* = 0.00037 and *P* = 0.73, respectively). It should be noted, however, that the Icelandic cystatin C sample set is very small (N = 284) and possibly underpowered to replicate the reported association with the *STC1* SNP. The *STC1* SNP did show association with SCr in our dataset (*P* = 1.6·10^−6^) as was observed in the analysis by Köttgen et al [14]. The *SHROOM3* and *GATM-SPATA5L1* SNPs showed suggestive association with CKD in the analysis by Köttgen et al [14] and our data support this association but do not constitute a conclusive replication. In contrast to the *UMOD* variant, we did not observe an interaction between the variants at these other loci and age. Furthermore, none of the SNPs did associate with kidney stones in Iceland (data not shown). Finally, Köttgen et al [14] reported suggestive association between eGFRcrea and variants at *JAG1* (GeneID: 182); we did not replicate this finding in our SCr scan (for rs6040055-T: effect = −0.27, 95% CI = −0.60−0.05), *P* = 0.17).

**Table 4:** Replication of loci recently found to associate with eGFRcrea and eGFRcys.

Gene		*UMOD*		*SHROOM3*		*GATM -SPATA5L1*		*STC1*		*CST3-CST9*	
SNP-Allele		rs4293393-T		rs17319721-A		rs2467853-G		rs1731274-G		rs13038305-T	
Trait		Association	Age effect	Association	Age effect	Association	Age effect	Association	Age effect	Association	Age effect
CKD#	OR	0.77	1.003	1.06	0.998	1.04	1.001	1.06	1.001	0.97	1.002
N = 2,944/	95% CI	(0.72,0.83)	(0.999,1.008)	(1.01,1.12)	(0.994,1.001)	(0.98,1.09)	(0.997,1.004)	(1.00,1.11)	(0.997,1.004)	(0.91,1.04)	(0.998,1.006)
35,502	P	2.6·10^−10^	0.19	0.044	0.27	0.26	0.65	0.080	0.69	0.5	0.38
SCr	Effect	−1.81	−0.079	0.69	0.007	0.55	−0.004	0.94	0.009	−0.09	−0.027
N = 21,952	95% CI	(−2.2,−1.42)	(−0.100,−0.058)	(0.37,1.00)	(−0.01,0.025)	(0.22,0.87)	(−0.022,0.013)	(0.62,1.25)	(−0.008,0.0260)	(−0.47,0.29)	(−0.048,−0.006)
	P	8.4·10^−14^	1.3·10^−9^	0.00057	0.49	0.0067	0.69	1.6e-06	0.41	0.71	0.037
Cystatin C	Effect	−0.22	0.011	0.06	−0.003	0.03	0.006	0.06	−0.011	−0.71	−0.001
N = 284	95% CI	(−0.67,0.22)	(−0.018,0.041)	(−0.28,0.4)	(−0.026,0.019)	(−0.32,0.39)	(−0.018,0.03)	(−0.29,0.42)	(−0.035,0.012)	(−1.09,−0.32)	(−0.028,0.025)
	P	0.32	0.46	0.72	0.77	0.85	0.63	0.73	0.34	0.00037	0.92

We tested the loci recently identified by Köttgen et al [14] to associate with eGFRcrea and eGFRcys for association with chronic kidney disease (CKD), and serum concentrations of creatinine (SCr) and cystatin C in our imputed Icelandic datasets. SCr effects are given in µmol/L and cystatin C effects are given in mg/L. The association analysis for all four SNPs was performed using expected allele counts from the IMPUTE software [34].

# For CKD, the sample size was 2,944 cases and 35,502 controls. The allele frequencies of rs17319721-A, rs2467853-G, rs1731274-G, and rs13038305-T in Iceland are 0.832, 0.719, 0.905, and 0.438, respectively.

In summary, we describe sequence variants next to and in *UMOD* that associate with increased risk of CKD and elevated SCr but confer protection against kidney stones. We also demonstrate an interaction between these variants and both age and comorbid conditions that are related to decline in kidney function. Our observations indicate that *UMOD* is important for maintaining kidney function with advancing age and exposure to risk factors that are associated with aging, such as HTN, type 2 DM and cardiovascular disease.

## Materials and Methods

### Study subjects from Iceland

Landspitali University Hospital (LUH) is a regional hospital for the greater Reykjavík area and a tertiary referral center for the entire Icelandic nation. The population of Iceland is comprised of 330,000 inhabitants of whom approximately 200,000 reside in the greater Reykjavik area. The nation's only nephrology clinic is located at LUH and all laboratory tests for the primary care clinics of the greater Reykjavik area are performed in the hospital's laboratories. We obtained results of all SCr measurements performed during the period 2003 to 2008 from the computerized database of the Clinical Laboratories at LUH and used the SCr values to identify those with chronic kidney disease (CKD) based on calculation of the estimated glomerular filtration rate (eGFR) by the original 4-variable Modification of Diet in Renal Disease (MDRD) study equation. We classified those with eGFR<60 ml/min/1.73 m^2^ as having CKD. All individuals with acute kidney injury and those who had eGFR<60 ml/min/1.73 m^2^ of less than 3 months duration were excluded from the CKD sample set. The study included CKD patients that had donated blood through various genetic programs at deCODE genetics.

Biochemical measurements including SCr, serum urea, serum uric acid and serum cystatin C were available from two laboratories, the Laboratory in Mjodd, Reykjavik, Iceland, a private outpatient laboratory, and the Clinical Biochemistry Laboratory of LUH, serving both inpatients and outpatients. The main referral center for the Laboratory in Mjodd is a multispecialty medical clinic in Reykjavik (Laeknasetrid). The laboratory tests were done in the years 1997–2008 at the Laboratory in Mjodd and in the years 2003–2008 at LUH. The Icelandic SCr measurements came from both laboratories, the Laboratory in Mjodd (N = 10,260) and LUH (N = 22,898, of whom 8,523 also had measurements from the Laboratory in Mjodd). At the LUH, the same enzymatic method was used for measurement of SCr during the study period (Vitros 950 Autoanalyzer, Ortho Clinical Diagnostics, Rochester, MN, USA), whereas at the Laboratory in Mjodd, SCr measurements were performed by modified kinetic Jaffe rection assays until May 2007 when an enzymatic method was introduced.

The Icelandic kidney stone cases consisted of patients with confirmed radiopaque kidney stones from the Icelandic Kidney Stone Disease Registry at LUH. The study included kidney stone patients that had donated blood through various genetic programs at deCODE genetics.

The coronary artery disease [29], stroke [30] and type 2 DM [31], [32] patient groups from Iceland have been described previously. The HTN sample set includes individuals who have been recruited into various genetic programs at deCODE and have: (1) self-reported HTN; (2) received the diagnosis of HTN at discharge from the LUH; or (3) have attended the Hypertension Clinic at LUH. The gout sample set includes subjects who were recruited into various genetic programs at deCODE and reported the use of either one of two specific anti-gout medications, allopurinol or colchicine.

The Icelandic controls used in the case-control genome-wide association studies (GWAS) and replication studies were selected among individuals who had participated in the various genetic programs at deCODE genetics; tremor, preeclampsia, endometriosis, psoriasis, type 2 DM, Alzheimer's disease, osteoarthritis, schizophrenia, peripheral artery disease, abdominal aortic aneurysm, chronic obstructive pulmonary disease, stroke, osteoporosis, coronary artery disease, HTN, asthma, Parkinson's disease, sleep apnea, age-related macular degeneration, polycystic ovary syndrome, rheumatoid arthritis, lung cancer, longevity, benign prostatic hyperplasia, enuresis, migraine, glaucoma, attention deficit hyperactivity disorder, prostate cancer, infectious diseases, anxiety, expression studies, autism, dyslexia, melanoma, colorectal cancer, deep vein thrombosis, restless leg syndrome, studies on addiction and population controls. Individuals who reported a history of the trait being tested (e.g. CKD) were excluded from the control set. Some of the controls participated in more than one genetic program in which case their genotypes are only included once.

The study was approved by the Icelandic Data Protection Authority and the National Bioethics Committee. All patients signed informed consent and donated blood samples. Personal identities of the patients and biological samples were encrypted by a third party system provided by the Icelandic Data Protection Authority.

### Study subjects from The Netherlands

All samples with SCr measurements came from the Nijmegen Biomedical Study. The details of this study have been reported previously [33]. Briefly, this is a population-based survey conducted by the Department of Epidemiology and Biostatistics and the Department of Clinical Chemistry of the Radboud University Nijmegen Medical Center (RUNMC), in which 9,371 individuals participated from a total of 22,500 age- and sex-stratified, randomly selected inhabitants of Nijmegen. The subjects were invited to participate in a study on gene-environment interactions in complex diseases. All participants filled out a questionnaire on lifestyle and medical history at baseline and 6500 of them donated blood samples for DNA isolation and biochemical studies. A fraction of the study participants were previously genotyped with the Illumina HumanHap300 or HumanHapCNV370 bead chips; these were selected to serve as controls in GWAS on prostate and breast cancer and were selected primarily based on age. A total of 1,819 individuals had both serum creatinine measurements and genome-wide SNP data available for analysis in this study.

The Dutch patients with kidney stones were recruited from two sources: The outpatient clinics of the RUNMC and The Nijmegen Biomedical Study. All patients who present to the outpatient clinics of the RUNMC are invited to participate in a study on the effects of genes and lifestyle on the development of urological diseases. In case of consent, the patients fill out a questionnaire on lifestyle and donate a blood sample for DNA isolation. The controls for the analysis of kidney stone disease were also taken from the biobank of the Nijmegen Biomedical Study. All patients and controls were of self-reported European descent and were fully informed about the goals and the procedures of these studies. The study protocols for the recruitment of patients from outpatient clinics and the recruitment of participants to the Nijmegen Biomedical Study were approved by the RUNMC Institutional Review Board. All study subjects gave written informed consent.

### Illumina genome-wide genotyping

All Icelandic case and control samples were assayed with the Illumina HumanHap300 or HumanHapCNV370 bead chips (Illumina, SanDiego, CA, USA), containing 317,503 and 370,404 haplotype tagging SNPs derived from phase I of the International HapMap project, respectively. Only SNPs present on both chips were included in the analysis and SNPs were excluded if they had: (a) yield lower than 95% in cases or controls; (b) minor allele frequency less than 1% in the population; or (c) showed significant deviation from Hardy-Weinberg equilibrium in the controls (P<0.001). Any samples with a call rate below 98% were excluded from the analysis. The final analysis included 302,379 SNPs.

### Imputation of SNP genotypes

The genome-wide association scan was based on expected allele counts obtained with the IMPUTE software [34], using the HapMap CEU samples as a training set [15]. The test for association was then performed using the expected allele counts as covariates. The imputation information was estimated by the ratio of the observed likelihood of allele counts and the likelihood of allele counts assuming perfect information under the assumption of Hardy-Weinberg equilibrium. The estimated information for the four SNPs imputed in Table 4 was high in all instances (>0.96).

### Single SNP genotyping

Single SNP genotyping of all samples was carried out at deCODE genetics in Reykjavik, Iceland, applying the same platform to all populations studied. All single SNP genotyping was carried out using the Centaurus (Nanogen) platform [35]. The quality of each Centaurus SNP assay was evaluated by genotyping each assay on the CEU samples and comparing the results with the HapMap data. All assays had a mismatch rate <0.5%. Additionally, all markers were re-genotyped on more than 10% of samples typed with the Illumina platform resulting in an observed mismatch in less than <0.5% of samples.

### Association analysis

For case-control association analysis, e.g. for CKD and kidney stones, we utilized a standard likelihood ratio statistic, implemented in the NEMO software [32] to calculate two-sided *P* values and odds ratios (ORs) for each individual allele, assuming a multiplicative model for risk, i.e. that the risk of the two alleles carried by a person multiplies [36]. Allelic frequencies, rather than carrier frequencies, are presented for the markers and *P* values are given after adjustment for the relatedness of the subjects. When estimating genotype specific OR, genotype frequencies in the population were estimated assuming Hardy-Weinberg equilibrium.

Results from multiple case-control groups were combined using a Mantel-Haenszel model [37] in which the groups were allowed to have different population frequencies for alleles, haplotypes and genotypes but were assumed to have common relative risks.

For the quantitative trait association analysis, e.g. for SCr and cystatin C, we utilized a robust linear regression based on an M estimator [38] as implemented in the rlm function of the R software package [39]. An additive model for SNP effects was assumed in all instances. All associations with quantitative traits were performed adjusting for age and sex.

### Estimation and testing of interaction effects

Interaction effects were tested by assuming all main effects and lower order interaction effects were present under the null model but not the interaction effect, resulting in a one degree of freedom model. For example, when testing for an interaction effect on SCr between age and the rs4293393-T allele count, the null model included as covariates age, sex and the rs4293393-T allele count. The alternative model included all these covariates as well as the product of the interaction of age and the rs4293393-T allele counts. Similarly, when testing for the interaction between age, the number of comorbid conditions and the rs4293393-T allele count, the null model included as covariates age, sex, rs4293393-T allele count, the product of interaction of age and rs4293393-T allele count, the product of interaction of the number of comorbid conditions and rs4293393-T allele count, and the product of interaction of age and the number of comorbid conditions and the alternative model added the product of interaction of age, the rs4293393-T allele count and the number of comorbid conditions. In the instances when an interaction effect was estimated, the main effect estimates and *P* values shown were obtained from fitting the appropriate model without an interaction effect.

### Correction for relatedness of the subjects and genomic control

Some of the individuals in the Icelandic patient and control groups are related to each other, causing the chi-square test statistic to have a mean >1 and median >0.675. We estimated the inflation factor for the genome-wide association by calculating the average of the 302,379 chi-square statistics, which was a method of genomic control [40] to adjust for both relatedness and potential population stratification. The inflation factor was estimated as 1.15 for CKD and 1.22 for SCr and all the results presented from association with these traits were adjusted based on these inflation factors.

## Supporting Information

Figure S1 QQ plot of 2.5 million SNPs in the genome-wide association scans for chronic kidney disease. The black dots represent the observed P values and the blue ‘x’s represent the P values scaled down by an inflation factor estimated using genomic control (1.15). The diagonal red line represents where the dots are expected to fall under the null hypothesis of no association. The horizontal green line represents the threshold for genome-wide significance.
(0.69 MB TIF)

Figure S2 QQ plot of 2.5 million SNPs in the genome-wide association scans for serum creatinine. The black dots represent the observed P values and the blue ‘x’s represent the P values scaled down by an inflation factor estimated using genomic control (1.22). The diagonal red line represents where the dots are expected to fall under the null hypothesis of no association. The horizontal green line represents the threshold for genome-wide significance.
(0.69 MB TIF)

Figure S3 The observed distribution of SCr measurements in Iceland and the Netherlands. The measurements from Iceland come from the Laboratory in Mjodd and the Clinical Biochemistry Laboratory of Landspitali University Hospital (LUH). The red line denotes the population median and the two dashed blue lines the 5% and 95% quantiles. Measurements above 200 are lumped together for visualization purposes. The unit of measurement is µmol/L.
(0.05 MB EPS)

Table S1 Strongest SNP associations (P<2·10^−5^) with CKD outside the *UMOD* region on chromosome 16p12.
(0.05 MB DOC)

Table S2 Strongest SNP associations (P<10^−6^) with SCr outside the *UMOD* region on chromosome 16p12.
(0.10 MB DOC)

Table S3 Association of rs4293393-T with risk factors of kidney function decline in Icelandic case-control groups.
(0.03 MB DOC)

Table S4 Results of age-specific association for rs4293393-T and kidney stones using year of birth (YOB) as a proxy for age at onset.
(0.03 MB DOC)
