# Correction: Association of Variants at *UMOD* with Chronic Kidney Disease and Kidney Stones—Role of Age and Comorbid Diseases

**DOI:** 10.1371/annotation/8e7ba8d6-a174-4a3a-93b4-510d5ca7ed1e

**Published:** 2010-11-02

**Authors:** Daniel F. Gudbjartsson, Hilma Holm, Olafur S. Indridason, Gudmar Thorleifsson, Vidar Edvardsson, Patrick Sulem, Femmie de Vegt, Frank C. H. d'Ancona, Martin den Heijer, Jack F. M. Wetzels, Leifur Franzson, Thorunn Rafnar, Kristleifur Kristjansson, Unnur S. Bjornsdottir, Gudmundur I. Eyjolfsson, Lambertus A. Kiemeney, Augustine Kong, Runolfur Palsson, Unnur Thorsteinsdottir, Kari Stefansson

An author was omitted from the published paper. Jack F. M. Wetzels should be listed as author number 10, affiliated with: Department of Nephrology, Radboud University Nijmegen Medical Center, Nijmegen, The Netherlands. Prof. Wetzels' contribution to the paper was: Performed the experiments. Prof. Wetzels declares no competing interests.

