# Supplementary figures and images for: Association of Variants at UMOD with Chronic Kidney Disease and Kidney Stones—Role of Age and Comorbid Diseases
Source: PLoS Genet. 2010 Jul 29;6(7):e1001039. doi: 10.1371/journal.pgen.1001039 (PMC2912386; doi:10.1371/journal.pgen.1001039)

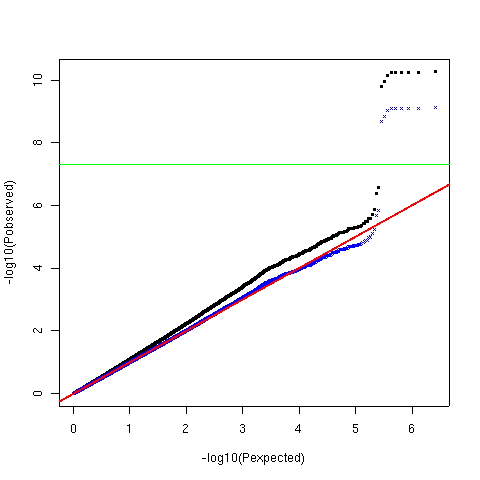

Supplement: Figure S1 — QQ plot of 2.5 million SNPs in the genome-wide association scans for chronic kidney disease. The black dots represent the observed P values and the blue ‘x’s represent the P values scaled down by an inflation factor estimated using genomic control (1.15). The diagonal red line represents where the dots are expected to fall under the null hypothesis of no association. The horizontal green line represents the threshold for genome-wide significance. (0.69 MB TIF) [file pgen.1001039.s001.tif]

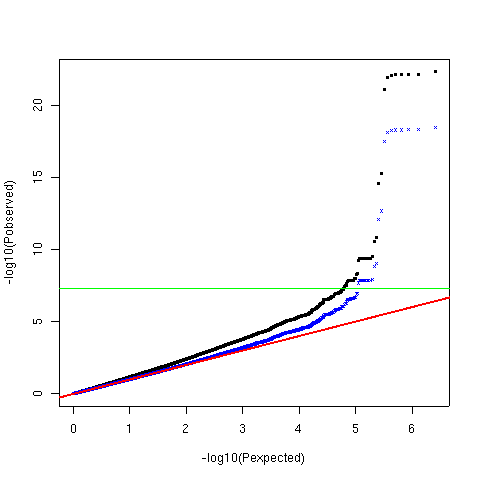

Supplement: Figure S2 — QQ plot of 2.5 million SNPs in the genome-wide association scans for serum creatinine. The black dots represent the observed P values and the blue ‘x’s represent the P values scaled down by an inflation factor estimated using genomic control (1.22). The diagonal red line represents where the dots are expected to fall under the null hypothesis of no association. The horizontal green line represents the threshold for genome-wide significance. (0.69 MB TIF) [file pgen.1001039.s002.tif]
