# Supplementary material for: Association of Variants at UMOD with Chronic Kidney Disease and Kidney Stones—Role of Age and Comorbid Diseases
Source: PLoS Genet. 2010 Jul 29;6(7):e1001039. doi: 10.1371/journal.pgen.1001039 (PMC2912386; doi:10.1371/journal.pgen.1001039)
Supplement: Table S1 — Strongest SNP associations (P<2·10−5) with CKD outside the UMOD region on chromosome 16p12. (0.05 MB DOC) [file pgen.1001039.s004.doc]

|  | **Effect allele/** |  |  | **N** |  |  |  |  |
| --- | --- | --- | --- | --- | --- | --- | --- | --- |
| **SNP** | **other** | **Chr** | **Position** | **Case/Ctrl** | **Freq** | **Info** | **OR (95%CI)** | **P** |
| rs2606414 | T/C | 1 | 2,857,008 | 2912/34796 | 0.28 | 0.99 | 0.86 (0.81,0.92) | 2.0∙10^-5^ |
| rs2842933 | G/A | 1 | 2,857,821 | 2912/34796 | 0.72 | 0.99 | 1.16 (1.08,1.24) | 2.0∙10^-5^ |
| rs17717776 | G/A | 2 | 107,142,652 | 2912/34796 | 0.16 | 0.80 | 0.76 (0.67,0.86) | 1.1∙10^-5^ |
| rs9288534 | G/A | 2 | 156,405,528 | 2912/34796 | 0.79 | 0.99 | 0.86 (0.80,0.92) | 1.6∙10^-5^ |
| rs9310709 | T/C | 3 | 23,068,578 | 2904/34775 | 0.59 | 1.00 | 0.87 (0.82,0.92) | 1.6∙10^-6^ |
| rs9835506 | G/C | 3 | 55,210,164 | 2912/34796 | 0.38 | 1.00 | 0.87 (0.82,0.93) | 1.2∙10^-5^ |
| rs10511174 | G/A | 3 | 100,745,793 | 2883/34539 | 0.05 | 1.00 | 1.33 (1.17,1.51) | 6.6∙10^-6^ |
| rs13070584 | T/C | 3 | 100,749,027 | 2912/34796 | 0.05 | 0.78 | 1.42 (1.23,1.65) | 2.3∙10^-6^ |
| rs10941694 | G/A | 5 | 45,233,536 | 2912/34796 | 0.94 | 0.76 | 0.74 (0.64,0.84) | 9.0∙10^-6^ |
| rs461189 | T/G | 13 | 31,437,019 | 2912/34796 | 0.38 | 0.99 | 0.87 (0.81,0.93) | 1.9∙10^-5^ |
| rs2773968 | T/A | 13 | 31,437,234 | 2912/34796 | 0.61 | 0.99 | 1.16 (1.08,1.23) | 1.5∙10^-5^ |
| rs169410 | T/C | 13 | 31,438,047 | 2912/34796 | 0.42 | 1.00 | 0.87 (0.82,0.93) | 1.5∙10^-5^ |
| rs203423 | T/C | 13 | 31,440,703 | 2912/34796 | 0.38 | 0.99 | 0.87 (0.81,0.93) | 1.9∙10^-5^ |
| rs9591069 | G/A | 13 | 46,976,461 | 2912/34796 | 0.11 | 0.98 | 0.81 (0.74,0.89) | 1.7∙10^-5^ |
| rs12908178 | G/A | 15 | 88,533,536 | 2912/34796 | 0.97 | 0.48 | 0.47 (0.33,0.66) | 1.9∙10^-5^ |
