# Supplementary material for: Association of Variants at UMOD with Chronic Kidney Disease and Kidney Stones—Role of Age and Comorbid Diseases
Source: PLoS Genet. 2010 Jul 29;6(7):e1001039. doi: 10.1371/journal.pgen.1001039 (PMC2912386; doi:10.1371/journal.pgen.1001039)
Supplement: Table S2 — Strongest SNP associations (P<10−6) with SCr outside the UMOD region on chromosome 16p12. (0.10 MB DOC) [file pgen.1001039.s005.doc]

| **SNP** | **Effect allele/**  **Other** | **Chr** | **Position** | **Freq** | **Info** | **Effect** | **P** |
| --- | --- | --- | --- | --- | --- | --- | --- |
| rs921197 | G A | 1 | 30,092,476 | 0.42 | 1.00 | -0.85 (-1.20, -0.50) | 1.5∙10^-6^ |
| rs4949498 | G A | 1 | 30,093,651 | 0.42 | 1.00 | -0.85 (-1.20, -0.51) | 1.4∙10^-6^ |
| rs1530677 | G A | 1 | 30,096,366 | 0.42 | 1.00 | -0.85 (-1.20, -0.51) | 1.4∙10^-6^ |
| rs6663901 | T C | 1 | 30,096,395 | 0.47 | 1.00 | 0.88 (0.53, 1.23) | 9.5∙10^-7^ |
| rs11694333 | G A | 2 | 41,938,866 | 0.60 | 0.99 | 1.26 (0.74, 1.78) | 1.9∙10^-6^ |
| rs7604595 | T C | 2 | 223,302,135 | 0.11 | 1.00 | -1.35 (-1.90, -0.80) | 1.6∙10^-6^ |
| rs4674686 | G A | 2 | 223,302,603 | 0.89 | 1.00 | 1.35 (0.80, 1.90) | 1.6∙10^-6^ |
| rs7564567 | G A | 2 | 223,302,616 | 0.89 | 1.00 | 1.35 (0.80, 1.90) | 1.6∙10^-6^ |
| rs4284825 | C A | 2 | 223,305,346 | 0.11 | 1.00 | -1.35 (-1.90, -0.80) | 1.6∙10^-6^ |
| rs2894472 | T G | 2 | 223,307,247 | 0.11 | 1.00 | -1.35 (-1.90, -0.80) | 1.6∙10^-6^ |
| rs10173577 | T C | 2 | 223,318,319 | 0.13 | 0.95 | -1.30 (-1.85, -0.76) | 2.4∙10^-6^ |
| rs1499989 | T C | 3 | 119,483,894 | 0.58 | 1.00 | -0.85 (-1.20, -0.50) | 1.6∙10^-6^ |
| rs316020 | G A | 6 | 160,589,071 | 0.87 | 1.00 | 1.24 (0.72, 1.75) | 2.4∙10^-6^ |
| rs316009 | T C | 6 | 160,595,754 | 0.13 | 1.00 | -1.24 (-1.75, -0.72) | 2.6∙10^-6^ |
| rs10480299 | T C | 7 | 151,036,751 | 0.74 | 1.00 | -0.96 (-1.35, -0.57) | 1.3∙10^-6^ |
| rs10480300 | T C | 7 | 151,036,938 | 0.26 | 1.00 | 0.96 (0.57, 1.34) | 1.4∙10^-6^ |
| rs7805747 | G A | 7 | 151,038,734 | 0.80 | 0.89 | -1.45 (-1.99, -0.90) | 2.2∙10^-7^ |
| rs10224210 | T C | 7 | 151,044,127 | 0.74 | 0.99 | -1.02 (-1.43, -0.62) | 7.2∙10^-7^ |
| rs10224002 | G A | 7 | 151,045,974 | 0.26 | 0.99 | 1.00 (0.60, 1.40) | 9.3∙10^-7^ |
| rs1547470 | T C | 7 | 151,063,802 | 0.47 | 1.00 | 0.90 (0.54, 1.26) | 8.9∙10^-7^ |
| rs885273 | G A | 7 | 151,067,285 | 0.55 | 1.00 | -0.89 (-1.24, -0.55) | 3.5∙10^-7^ |
| rs3758086 | G A | 8 | 23,770,937 | 0.58 | 1.00 | -0.91 (-1.25, -0.56) | 3.1∙10^-7^ |
| rs7007761 | T C | 8 | 23,775,516 | 0.42 | 1.00 | 0.93 (0.57, 1.28) | 2.8∙10^-7^ |
| rs6999484 | G A | 8 | 23,784,216 | 0.58 | 1.00 | -0.91 (-1.25, -0.56) | 2.8∙10^-7^ |
| rs11778129 | G A | 8 | 23,788,171 | 0.58 | 1.00 | -0.91 (-1.25, -0.56) | 2.8∙10^-7^ |
| rs4871905 | G C | 8 | 23,790,992 | 0.58 | 1.00 | -0.91 (-1.25, -0.56) | 2.8∙10^-7^ |
| rs9314273 | G C | 8 | 23,791,504 | 0.58 | 1.00 | -0.91 (-1.25, -0.56) | 2.8∙10^-7^ |
| rs819196 | T A | 8 | 23,798,666 | 0.47 | 1.00 | 0.91 (0.56, 1.26) | 2.9∙10^-7^ |
| rs10109414 | T C | 8 | 23,807,096 | 0.42 | 1.00 | 0.91 (0.56, 1.25) | 3.1∙10^-7^ |
| rs1731274 | G A | 8 | 23,822,264 | 0.45 | 1.00 | 0.84 (0.50, 1.19) | 1.7∙10^-6^ |
| rs17786744 | G A | 8 | 23,832,951 | 0.42 | 1.00 | 0.90 (0.55, 1.24) | 4.4∙10^-7^ |
| rs1705699 | T C | 8 | 23,837,398 | 0.54 | 1.00 | -0.85 (-1.19, -0.50) | 1.3∙10^-6^ |
| rs1629026 | C A | 8 | 23,842,017 | 0.56 | 1.00 | -0.88 (-1.24, -0.53) | 9.5∙10^-7^ |
| rs4871907 | C A | 8 | 23,842,729 | 0.43 | 1.00 | 0.88 (0.52, 1.25) | 1.7∙10^-6^ |
| rs17549749 | G A | 15 | 51,747,920 | 0.28 | 1.00 | -0.98 (-1.36, -0.59) | 6.7∙10^-7^ |
| rs150355 | T C | 16 | 22,724,922 | 0.93 | 1.00 | -1.68 (-2.36, -0.99) | 1.5∙10^-6^ |
