# Supplementary material for: Association of Variants at UMOD with Chronic Kidney Disease and Kidney Stones—Role of Age and Comorbid Diseases
Source: PLoS Genet. 2010 Jul 29;6(7):e1001039. doi: 10.1371/journal.pgen.1001039 (PMC2912386; doi:10.1371/journal.pgen.1001039)
Supplement: Table S3 — Association of rs4293393-T with risk factors of kidney function decline in Icelandic case-control groups. (0.03 MB DOC) [file pgen.1001039.s006.doc]

|  | **N** |  | **Frequency** | |  |  |
| --- | --- | --- | --- | --- | --- | --- |
| **Disease** | **Case** | **Ctrl** | **Case** | **Ctrl** | **OR (95% CI)** | ***P*** |
| Hypertension | 7,427 | 33,462 | 0.810 | 0.799 | 1.07 (1.01, 1.12) | 0.014 |
| MI | 3,661 | 36,912 | 0.807 | 0.801 | 1.04 (0.98, 1.11) | 0.22 |
| Stroke | 1,926 | 38,493 | 0.809 | 0.801 | 1.05 (0.97, 1.15) | 0.24 |
| T2DM | 1,909 | 38,493 | 0.797 | 0.801 | 0.97 (0.89, 1.06) | 0.57 |
