# Supplementary material for: Association of Variants at UMOD with Chronic Kidney Disease and Kidney Stones—Role of Age and Comorbid Diseases
Source: PLoS Genet. 2010 Jul 29;6(7):e1001039. doi: 10.1371/journal.pgen.1001039 (PMC2912386; doi:10.1371/journal.pgen.1001039)
Supplement: Table S4 — Results of age-specific association for rs4293393-T and kidney stones using year of birth (YOB) as a proxy for age at onset. (0.03 MB DOC) [file pgen.1001039.s007.doc]

|  |  | **Kidney stones** | |  |  |
| --- | --- | --- | --- | --- | --- |
| **Subjects** |  | **N** | **Freq** | **OR (95% CI)** | ***P*** |
| YOB≥1950 |  | 957 | 0.785 | 0.91 (0.81, 1.01) | 0.085 |
| 1950>YOB≥1940 |  | 539 | 0.781 | 0.88 (0.76, 1.02) | 0.10 |
| 1940>YOB≥1930 |  | 673 | 0.781 | 0.88 (0.77, 1.01) | 0.065 |
| 1930>YOB≥1920 |  | 566 | 0.782 | 0.89 (0.77, 1.03) | 0.11 |
| 1920>YOB |  | 181 | 0.773 | 0.85 (0.66, 1.09) | 0.19 |
| **Controls** |  | 40,253 | 0.801 | - | - |
